# Structure of Rubisco from *Arabidopsis thaliana* in complex with 2-carboxyarabinitol-1,5-bisphosphate

**DOI:** 10.1107/S2059798317017132

**Published:** 2018-01-01

**Authors:** Karin Valegård, Dirk Hasse, Inger Andersson, Laura H. Gunn

**Affiliations:** a Laboratory of Molecular Biophysics, Department of Cell and Molecular Biology, Uppsala University, Husargatan 3, Box 596, SE-751 24 Uppsala, Sweden

**Keywords:** ribulose-1,5-bisphosphate carboxylase/oxygenase, Rubisco, 2-carboxy­arabinitol-1,5-bisphosphate, carbon fixation, *Arabidopsis thaliana*, *rbcS* multigene family, isoforms, merohedral twinning

## Abstract

The crystal structure of ribulose-1,5-bisphosphate carboxylase/oxygenase (Rubisco) from *Arabidopsis thaliana* is reported at 1.5 Å resolution. In light of the importance of *A. thaliana* as a model organism for understanding higher plant biology, and the pivotal role of Rubisco in photosynthetic carbon assimilation, there has been a notable absence of an *A. thaliana* Rubisco crystal structure. *A. thaliana* Rubisco is an L_8_S_8_ hexadecamer comprising eight plastome-encoded catalytic large (L) subunits and eight nuclear-encoded small (S) subunits. *A. thaliana* produces four distinct small-subunit isoforms (RbcS1A, RbcS1B, RbcS2B and RbcS3B), and this crystal structure provides a snapshot of *A. thaliana* Rubisco containing the low-abundance RbcS3B small-subunit isoform. Crystals were obtained in the presence of the transition-state analogue 2-carboxy-d-arabinitol-1,5-bisphosphate. *A. thaliana* Rubisco shares the overall fold characteristic of higher plant Rubiscos, but exhibits an interesting disparity between sequence and structural relatedness to other Rubisco isoforms. These results provide the structural framework to understand *A. thaliana* Rubisco and the potential catalytic differences that could be conferred by alternative *A. thaliana* Rubisco small-subunit isoforms.

## Abstract 2

The first crystal structure of Rubisco from *A. thaliana* is described and is compared with all other form I Rubisco crystal structures. This new structure is used to discuss the catalytic differences that could be conferred by alternative Rubisco small-subunit isoforms, and the potential benefit of differential expression of such isoforms on photosynthetic carbon assimilation in land plants.

## 1. Introduction   

Ribulose-1,5-bisphosphate carboxylase/oxygenase (Rubisco) catalyses the addition of carbon dioxide to ribulose 1,5-bisphosphate (RuBP) in the first step of the photosynthetic Calvin–Benson–Bassham cycle. However, molecular oxygen competes with carbon dioxide for addition to RuBP, which results in photorespiration: the production of a toxic compound, the recycling of which consumes energy and releases fixed CO_2_. Poor specificity (*S*_c/o_) for substrate carbon dioxide, along with a slow catalytic turnover rate, means that Rubisco often limits the growth rate of higher plants (Long *et al.*, 2006 ▸; Andersson, 2008 ▸).

Rubisco catalysis occurs at the interface of two ∼55 kDa large subunits (LSu; encoded by the *rbc*L gene in the plastome). Rubisco in higher plants also contains ∼15 kDa small subunits (SSu; *rbc*S gene, nuclear-encoded), which are produced in the cytosol as precursor SSus with an N-terminal transit peptide. After transport through the chloroplast envelope, the transit peptide of the precursor SSu is cleaved by a stromal peptidase, producing the mature SSu (Jarvis & Soll, 2001 ▸). Within the chloroplast, four L_2_ units assemble into an L_8_ core, which is capped at each end by a tetrad of SSus, yielding an ∼550 kDa L_8_S_8_ hexadecameric enzyme.

The nucleus of higher plants encodes an *rbc*S multigene family that may provide the opportunity for differential SSu expression in response to temperature, tissue type, developmental stage and light treatment (Eilenberg *et al.*, 1991 ▸; Wanner & Gruissem, 1991 ▸; Dedonder *et al.*, 1993 ▸; Meier *et al.*, 1995 ▸; Ewing *et al.*, 1998 ▸; Yoon *et al.*, 2001 ▸; Suzuki *et al.*, 2009 ▸). The functional requirement for differential SSu expression remains unclear, but may be linked to the potential influence that SSus can have on Rubisco catalysis and L_8_S_8_ content (Du *et al.*, 2000 ▸).

The number of SSu isoforms produced in different species varies greatly. For example, the nuclei of rice (Suzuki *et al.*, 2009 ▸) and wheat (Smith *et al.*, 1983 ▸) have five and 12 *rbc*S gene copies, respectively. The model plant species *Arabidopsis thaliana* encodes four SSu isoforms: *rbc*S1B, *rbc*S2B and *rbc*S3B are closely located on chromosome 5, while *rbc*S1A is encoded by chromosome 1 (Niwa *et al.*, 1997 ▸). *A. thaliana* SSu genes are differentially controlled by light and developmental cues (Brusslan & Tobin, 1992 ▸; Dedonder *et al.*, 1993 ▸). *A. thaliana* Rubisco SSus may contribute additively to provide sufficient Rubisco accumulation in leaves, and different isoforms do not alter photosynthesis under the present atmospheric CO_2_ partial pressures (Izumi *et al.*, 2012 ▸) and do not confer differential kinetic properties at 25°C (Atkinson *et al.*, 2017 ▸). However, the potential kinetic contribution of alternative SSu isoforms under more extreme environmental conditions has not been fully explored; Rubisco with higher specificity for substrate carbon dioxide may in fact be produced at higher temperatures in *A. thaliana* (Cavanagh, 2016 ▸).

Despite high homology in sequence and the overall holo­enzyme structure, there is significant variation in the kinetic performances of form I Rubiscos. Understanding the underlying structural basis that confers the kinetic properties of Rubisco is of great importance to inform Rubisco engineering strategies in order to tailor plants suitable for future environmental conditions (Parry *et al.*, 2013 ▸). Despite not contributing residues to the Rubisco active site, it is clear that the Rubisco SSu exerts some long-range catalytic influence on holoenzyme performance (Spreitzer, 2003 ▸; van Lun *et al.*, 2011 ▸). The Rubisco SSu is essential for maximal activity (Andrews, 1988 ▸). Chimeric Rubisco enzymes that incorporate the SSu from another species exhibit kinetic properties that are more reminiscent of the donor SSu Rubisco (Sharwood *et al.*, 2008 ▸; Zhang *et al.*, 2011 ▸; Ishikawa *et al.*, 2011 ▸). Similarly, point mutations within the SSu can alter Rubisco kinetics (Kostov *et al.*, 1997 ▸; Spreitzer *et al.*, 2001 ▸; Genkov & Spreitzer, 2009 ▸).

Tight coordination of a large number of molecular products is required to regulate the transcription, translation, folding and assembly of Rubisco (Bracher *et al.*, 2017 ▸). Several *A. thaliana* Rubisco chaperone proteins have been structurally characterized, including RbcX (Kolesinski *et al.*, 2013 ▸), Rubisco activase (Hasse *et al.*, 2015 ▸), CbbY (Bracher *et al.*, 2015 ▸) and Rubisco assembly factor 1 (Hauser *et al.*, 2015 ▸). However, the structure of *A. thaliana* Rubisco has not been solved.

Here, we describe the crystal structure of the activated form of Rubisco from *A. thaliana* at 1.5 Å resolution with a bound transition-state analogue, 2-carboxy-d-arabinitol 1,5-bisphos­phate (2-CABP). These data provide a structural context for interactions between the LSu and SSus of *A. thaliana* Rubisco.

## 2. Materials and methods   

### 2.1. Plant material and growth conditions   

*Arabidopsis* (*A. thaliana* Col-0) seeds were sterilized in 20%(*v*/*v*) commercial bleach and 0.1% Tween 20, and washed extensively with sterile water. Sterilized seeds were incubated at 4°C for 3 d before sowing in commercial soil (ICA Garden, Sweden) and were grown at 20°C and ambient CO_2_ under 120–150 µmol photons m^−2^ s^−1^ in 12:12 h light:dark cycles.

### 2.2. Protein purification   

Rubisco was purified from *A. thaliana* plants. Freshly harvested leaves were homogenized in 100 ml 100 m*M* Bicine buffer pH 8.5 containing 10 m*M* MgCl_2_, 10 m*M* NaHCO_3_, 1 m*M* EDTA and 10 m*M* β-mercaptoethanol using a Bamix stick blender. The homogenate was cleared by centrifugation for 30 min at 40 000*g* and 4°C. The supernatant was filtered through a 1.2 µm syringe filter and applied onto a Superdex 200 column (26/60, GE Healthcare). Fractions containing Rubisco were identified by SDS–PAGE (data not shown) and applied onto a Mono Q column (10/100 GL, GE Healthcare). The column was washed with 100 m*M* Bicine buffer pH 8.5, 100 m*M* NaCl, 10 m*M* MgCl_2_, 10 m*M* NaHCO_3_, 1 m*M* EDTA, 10 m*M* β-mercaptoethanol before elution of Rubisco using a gradient of 100–500 m*M* NaCl with a gradient length of 120 ml in 100 m*M* Bicine buffer pH 8.5, 10 m*M* MgCl_2_, 10 m*M* NaHCO_3_, 1 m*M* EDTA, 10 m*M* β-mercaptoethanol. Peak fractions, as identified by SDS–PAGE (data not shown), were pooled and the buffer was changed to 100 m*M* HEPES pH 8.5, 10 m*M* MgCl_2_, 10 m*M* NaHCO_3_, 1 m*M* EDTA using a Vivaspin 100 000 MWCO concentrator (Vivascience). The protein was then incubated at room temperature for 30 min.

### 2.3. Crystallization and data collection   

Crystals of activated *A. thaliana* Rubisco in complex with the transition-state analogue 2-CABP (at a ratio of four times the concentration of Rubisco active sites) were obtained at 20°C by the sitting-drop vapour-diffusion method after mixing equal volumes of protein solution (10 mg ml^−1^) and reservoir solution from well D10 [5%(*w*/*v*) polyglutamic acid, 15%(*w*/*v*) PEG 4000, 100 m*M* sodium cacodylate pH 6.5] of The PGA Screen (Molecular Dimensions; Hu *et al.*, 2008 ▸). The droplets were equilibrated against the reservoir solution, and crystals appeared after a few days. Prior to data collection, crystals were transferred into a cryoprotectant solution [25%(*w*/*v*) ethylene glycol, 100 m*M* HEPES pH 8.5, 100 m*M* NaCl, 10 m*M* MgCl_2_, 10 m*M* NaHCO_3_, 20%(*w*/*v*) PEG 4000] and flash-cooled in liquid nitrogen.

Data were collected at 100 K using a Pilatus 6M detector on beamline ID-29 of the European Synchrotron Radiation Facility (ESRF), Grenoble, France.

### 2.4. Structure determination and refinement   

Diffraction data were processed and scaled using the *XDS* program package (Kabsch, 2010 ▸; Table 1 ▸), with 5% of the reflections set aside to calculate the quality factor *R*_free_ (Brünger, 1992 ▸). The structure of *A. thaliana* Rubisco was determined to 1.5 Å resolution by molecular replacement using *Phaser* (McCoy *et al.*, 2007 ▸) within the *CCP*4 software package (Winn *et al.*, 2011 ▸). The search model consisted of an L_2_S_2_ unit of activated spinach Rubisco (PDB entry 8ruc; Andersson, 1996 ▸). The *A. thaliana* Rubisco crystals are merohedrally twinned and twin refinement was performed using a combination of *REFMAC*5 (Murshudov *et al.*, 2011 ▸) and *PHENIX* (Adams *et al.*, 2010 ▸; Afonine *et al.*, 2012 ▸) interspersed with manual rebuilding using *O* (Jones *et al.*, 1991 ▸). Occupancy refinement was performed in *PHENIX*. The structures were evaluated using the wwPDB Validation Server (Berman *et al.*, 2003 ▸). Refinement statistics are presented in Table 1 ▸. The coordinates and structure factors have been deposited in the PDB with accession code 5iu0.

### 2.5. Sequence and structure comparison   

Pairwise structural alignments were performed with the least-squares superposition function in *O* using the default distance cutoff limit of 3.8 Å. Amino-acid sequence alignments were first created using *ClustalOmega* (Sievers *et al.*, 2011 ▸) before manual adjustment to match the structural alignments obtained in *O*. The graphical output was created in *ESPript* (Robert & Gouet, 2014 ▸).

### 2.6. Other software   

All figures displaying protein structures were prepared with the *PyMOL* Molecular Graphics System (v.1.7.4; Schrödinger).

## 3. Results   

### 3.1. Overall Rubisco structure   

**Figure 1:**
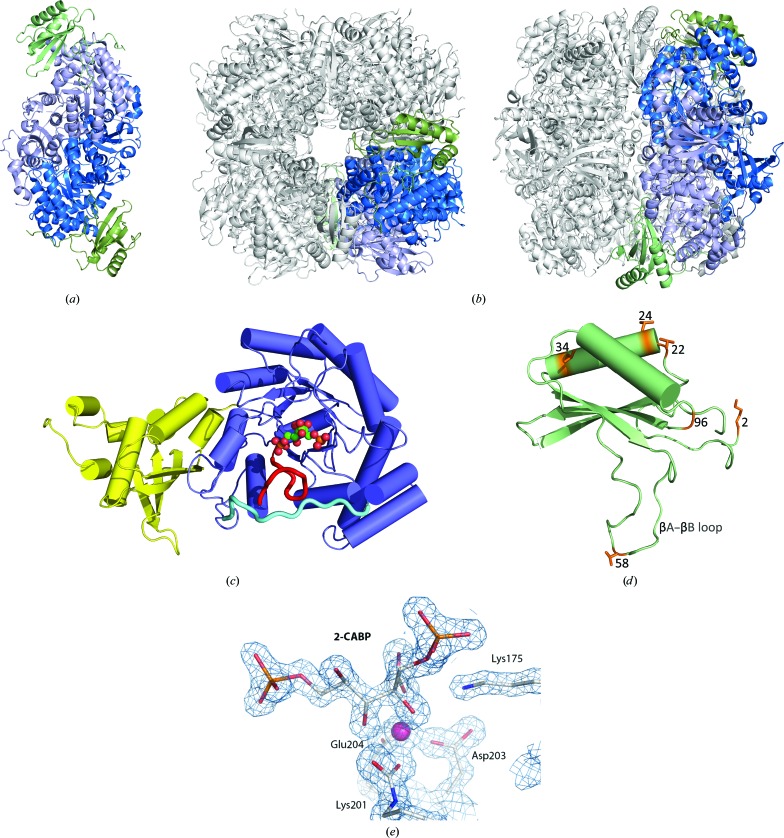
Three-dimensional crystal structure of *A. thaliana* Rubisco. (*a*) The L_2_S_2_ asymmetric unit of *A. thaliana* Rubisco. LSus are shown in shades of blue and SSus in green. (*b*) Top (left) and side (right) views of the overall hexadecameric (L_8_S_8_) structure of *A. thaliana* Rubisco. One asymmetric unit is shaded as depicted in (*a*), with the rest of the assembly shaded grey. (*c*) Structure of the LSu, with the N-terminal domain, C-terminal domain, loop 6 and C-terminal extension shown in yellow, blue, red and cyan, respectively. One 2-CABP molecule is shown bound at the active site. (*d*) Structure of the SSu, with residues that vary between different *A. thaliana* Rubisco SSu isoforms shown as orange sticks and numbered according to the mature peptide sequence. (*e*) 2-CABP is bound at the active site within well defined density. The Mg^2+^ ion that stabilizes the carbamate formed at the catalytic lysine is shown in pink.

*A. thaliana* Rubisco crystallized in the tetragonal space group *I*4, with unit-cell parameters *a* = 73.9, *b* = 88.2, *c* = 421.6 Å (Table 1 ▸). The crystals were merohedrally twinned, with a twin fraction of 0.48 (twin operator −*h*, *k*, −*l*). The crystallographic asymmetric unit contains a quarter of the L_8_S_8_ hexadecameric *A. thaliana* Rubisco complex, L_2_S_2_ (Fig. 1 ▸ *a*), with a crystal solvent content of 46% (*V*_M_ = 2.28 Å^3^ Da^−1^), where the molecular weight was estimated from the combined number of residues (604) from one LSu and one SSu (Matthews, 1968 ▸). Clear electron density was observed for residues 13–475 of the 479-amino-acid LSu and residues 1–123 of the 125-amino-acid SSu.

The *A. thaliana* Rubisco LSu exhibits the conventional LSu fold, comprising an N-terminal domain (residues 1–150) and a C-terminal domain (residues 151–479) (Fig. 1 ▸ *c*). The core of the LSu N-terminal domain is comprised of a four-stranded β-sheet and two α-helices, and the C-terminal domain contains an eight-stranded βα-barrel unit. Four conserved residues within the N-domain (Tyr20, Glu60, Thr65 and Asn123) contribute to the formation of an active site, together with residues in the loops of the C-terminal barrel domain in the adjacent LSu (Lys175, Lys177, Lys201, Asp203, Glu204, His294, Arg295, His327, Lys334 and Leu335) (Andersson, 2008 ▸; Kannappan & Gready, 2008 ▸; Supplementary Fig. S1).

The *A. thaliana* Rubisco structure is in the ‘activated state’, in which a carbamate formed at the catalytic lysine residue (Lys201) is stabilized by Mg^2+^ (Lorimer *et al.*, 1976 ▸). The ligand 2-CABP binds in a stoichiometric and almost irreversible manner to each activated catalytic site in Rubisco (Pierce *et al.*, 1980 ▸), and can be visualized in this structure in well defined density (Fig. 1 ▸ *e*). Unlike the substrate RuBP, 2-CABP does not turn over and thus the otherwise flexible loop 6 (the loop connecting β6 and α6 in Supplementary Fig. S1) folds over the ligand in this structure (Fig. 1 ▸ *c*, red). Loop 6 is further stabilized by residues within the LSu C-tail extension (Fig. 1 ▸ *c*, cyan).

The *A. thaliana* Rubisco SSu core consists of a four-stranded β-sheet and two α-helices (Fig. 1 ▸ *d*, Supplementary Fig. S2), an overall fold that is highly conserved in Rubisco SSus (Knight *et al.*, 1990 ▸). The length of the Rubisco SSu βA–βB loop, which extends into the solvent channel, varies greatly between Rubisco isoforms from different species (Knight *et al.*, 1990 ▸; Newman & Gutteridge, 1993 ▸; Taylor *et al.*, 2001 ▸). The *A. thaliana* Rubisco SSu βA–βB loop is 22 amino acids in length, which is characteristic of higher plant Rubisco SSus (Knight *et al.*, 1990 ▸; Figs. 1 ▸ *d* and 2 ▸, Supplementary Fig. S2).

### 3.2. Comparison with other Rubiscos   

The *A. thaliana* Rubisco structure was compared with all available L_8_S_8_ Rubisco structures in the PDB (Table 2 ▸). *A. thaliana* Rubisco exhibits high sequence identity to higher plant Rubiscos, with higher homology between the LSus (93–95%) than the SSus (72–76%; Table 2 ▸). Unsurprisingly, Rubisco subunit sequence similarity generally decreases with the evolutionary distance of the taxonomic group, and Rubisco enzymes within a taxonomic group show comparable levels of similarity. In general this tendency is also followed by the structure homology, as indicated by the root-mean-square deviations (r.m.s.d.s) between these Rubisco structures (Table 2 ▸). For instance, structural differences between LSus and SSus from crop plants are small, which is indicative of close kinship. However, in some cases there is a disparity between the sequence and structure homologies of the Rubisco subunits. Thus, whereas the *A. thaliana* Rubisco LSu sequence is very similar to that of rice Rubisco (94% amino-acid identity), which is reflected by a high structural resemblance between their LSus (r.m.s.d. of 0.25 Å), a significantly lower structural resemblance is observed in the SSus (r.m.s.d. of 1.20 Å), although the sequence similarity is only slightly lower (72% amino-acid identity) than those for the other crop plants (74–76% amino-acid identity). Analysis of the superimposed structures shows that this difference is mainly because of a single amino-acid deletion at position 46 of the rice Rubisco SSu (Supplementary Fig. S2), resulting in a tighter loop at this position. There are also some structural differences in the two C-terminal residues of the rice Rubisco SSu. Analysis of electron-density maps calculated for rice Rubisco shows that whereas there is well defined electron density for the loop around residues 46–47, there is only weak electron density for the SSu C-terminus, rendering the structure comparison in this region more uncertain. Similar tendencies as described here for Rubiscos from crop plants are also observed when other taxonomic groups are compared (Table 2 ▸) and, although interesting, these differences are small and are likely to be influenced by differences in crystal packing, resolution and refinement methods.

Phylogenetic analyses of Rubisco LSu sequences indicate that despite low bootstrap values for clades containing spinach, pea, tobacco and *A. thaliana* (34–67%), the rice Rubisco LSu diverges from these other higher plant Rubiscos with 99% bootstrap confidence (Supplementary Fig. S3). Thus, the *A. thaliana* LSu is phylogenetically distinct from rice Rubisco, despite these Rubiscos exhibiting the highest structural similarity to one another.

### 3.3. Capturing a low-abundance SSu   

**Figure 2:**
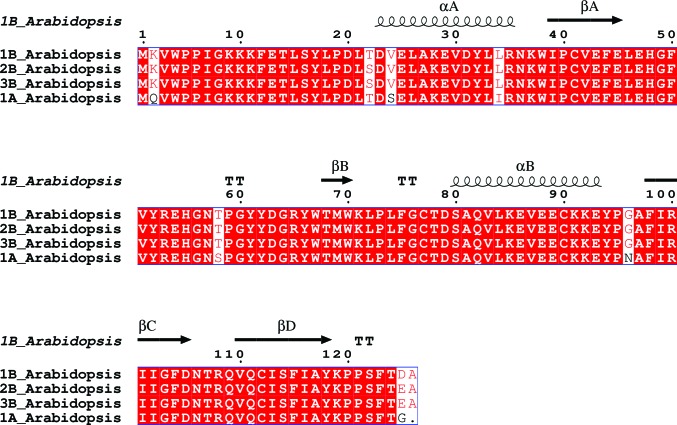
Sequences of the four Rubisco SSu isoforms from *A. thaliana*. The *A. thaliana* Rubisco structure presented in this study contains the RbcS1B isoform. Transit peptides were removed before analysis and residues are numbered relative to the mature peptide sequence. Conserved residues are boxed, strictly conserved residues have a red background and well conserved residues are shown in red letters. Gaps are represented by dots. Symbols above blocks of sequences annotate the Rubisco SSu secondary structure from PDB entry 5iu0: α, α-helix; β, β-strand; TT, strict β-turn. The secondary-structure elements were named αA, αB, βA, βB, βC and βD according to convention (Knight *et al.*, 1990 ▸). The sequence alignment was created using GenBank accession numbers BAB09355.1 (RbcS1B), AAO29974.1 (RbcS2B), AAL47390.1 (RbcS3B) and AEE34594.1 (RbcS1A).

The transit-peptide sequences differ between all four *A. thaliana* SSu isoforms (Supplementary Fig. S4), and the mature RbcS2B and RbcS3B protein sequences are identical (Fig. 2 ▸, Supplementary Fig. S4). Interpretation of the electron density indicates that the *A. thaliana* Rubisco SSu protein sequence in the structure presented in this study contains the RbcS1B isoform. The SSu amino-acid sequence from the structure differs from the RbcS1A SSu isoform at residues 2, 24, 34, 58 and 96 (Figs. 1 ▸ *d* and 2 ▸, Supplementary Fig. S4). There is only one amino-acid difference between RbcS1B and Rbcs2B/RbcS3B in the residues that were resolved in this structure: residue 22 is a serine in Rbcs2B/RbcS3B and is a threonine in RbcS1B and this structure. To confirm the identity of this residue, both threonine and serine (with a dual conformation) were separately modelled into the electron density before undergoing occupancy refinement. The resulting occupancies and difference maps were most consistent with this residue being threonine at full occupancy (data not shown). Furthermore, there was no mixture of residues at any given SSu amino acid in the structure, indicating that the Rubisco isoform that crystallized under these conditions was homogenous with respect to RbcS content.

### 3.4. Location of the amino-acid differences in *A. thaliana* SSu isoforms   

There are a total of eight sites that differ between the *A. thaliana* SSu isoforms at the mature peptide level (Supplementary Table S1, Fig. 1 ▸ *d*). The amino acids at several of these positions are close to LSu helix α8 in the C-terminal βα-barrel (Supplementary Fig. S1). Residues 22, 24 and 34 are in (or near) SSu helix αA (Fig. 2 ▸), which is proximal to LSu helix α8. Residue 2 (lysine in RbcSB SSus) is also located (i) within 5.5 Å of LSu helix α8 and (ii) within 3 Å of Glu454 in the ultimate LSu helix (helix αK; see Supplementary Fig. S1). Unlike the RbcS1A isoform, which encodes a glutamine at this position, Lys2 in the RbcSB family of SSu isoforms could influence holoenzyme structure–function *via* ionic interactions with Glu454 in the LSu C-tail extension that folds over and stabilizes loop 6 during catalysis (Knight *et al.*, 1990 ▸; Fig. 1 ▸ *c*).

The amino-acid difference between RbcS1A and the RbcSB family at residue 58 is minor (serine/threonine). This residue is in a strategic location at the apex of the βA–βB loop that extends into the solvent channel in form IA and IB Rubiscos, and is proximal to helix α3 in two different LSus. Residue 96 resides at the end of the SSu αB–βC loop. This hinge could be less flexible in RbcS1A (encoding asparagine) than in the RbcSB SSu family (encoding glycine). The Asn96 side chain in RbcS1A is likely to be located between the SSu C-terminus and the start of the SSu βA–βB loop. The last two SSu residues (124 and 125) were not resolved in this structure and are likely to be flexible.

## 4. Discussion   

### 4.1. Relevance of the Rubisco structure from *A. thaliana*   

*A. thaliana* is a model organism for research into photosynthesis in higher plants. *A. thaliana* was the first plant to have its genome sequenced, and a wealth of transcriptome data exists (Yamada *et al.*, 2003 ▸), making this organism amenable to functional genomics. The short generation time from seed to seed (approximately eight weeks), the diploid genome and the small plant size (allowing high-density growth) promote high-throughput research. Furthermore, *Agrobacterium tumefaciens*-facilitated nuclear transformation is well established for *A. thaliana* (Clough & Bent, 1998 ▸), and seed collections contain a multitude of individual gene-knockdown *A. thaliana* lines (http://www.arabidopsis.org), enabling efficient genetic manipulation and analyses.

It is surprising that the crystal structure of *A. thaliana* Rubisco had not been solved, given (i) the importance of *A. thaliana* for advancing our understanding of molecular plant biology and (ii) the central role of Rubisco in photosynthetic carbon fixation. The crystal structure presented in this study provides the structural framework to interpret *A. thaliana* Rubisco kinetics and interactions with accessory proteins, and adds to a growing database of higher plant Rubisco structures. A larger pool of sequence–structure data may provide us with greater power to try to understand natural Rubisco sequence–structure–function variation and how this could be harnessed in engineering strategies to enhance the kinetic performance of Rubisco enzymes.

### 4.2. The *A. thaliana* Rubisco structure is similar to those of other Rubiscos   

Despite differences in Rubisco primary amino-acid sequence, structural comparisons indicate that the overall secondary structure of the LSu in the various holoenzymes is highly conserved (Table 2 ▸), consistent with previous studies (Andersson & Backlund, 2008 ▸).

### 4.3. Capturing a ‘low-abundance’ SSu   

The electron density indicates that the SSu isoform in this structure is the RbcS1B isoform. Transcript levels of *rbc*S1B remain low under a wide range of tested environmental conditions, and *rbc*S1B is expected to represent only ∼8% of the total *rbc*S transcript pool in *A. thaliana* plants (Dedonder *et al.*, 1993 ▸; Izumi *et al.*, 2012 ▸; Atkinson *et al.*, 2017 ▸). Whether the *A. thaliana* plants grown for this experiment produced a higher proportion of Rubiscos containing the Rbcs1B isoform than previously reported cannot be answered because of a lack of transcript and protein information. It cannot be excluded that this Rubisco isoform was ‘titrated out’ during crystallization (meaning that the subpopulation of Rubisco L_8_ cores bound exclusively to RbcS1B SSus was preferentially crystallized, leaving behind any Rubisco complex that incorporated any other SSu isoform).

### 4.4. Homogenous SSu populations in crystals   

All SSu chains in the *A. thaliana* holoenzyme structure were the RbcS1B isoform. There was no ambiguity or mixture of amino acids at any position: electron density was distinct at all positions that differ between the isoforms. The Rubisco SSu population also appears to be homogenous in other Rubisco crystal structures. For example, the pea Rubisco structures deposited in the PDB as entries 4hhh (Loewen *et al.*, 2013 ▸) and 4mkv (M. C. Loewen, P. C. Loewen & J. Switala, unpublished work) contain different SSu peptide sequences to one another, but are consistent throughout each SSu within each structure. It remains unclear whether these data indicate that Rubisco containing RbcS1B was the predominant *A. thaliana* Rubisco population, that the SSu influences structure sufficiently that heterogenous populations cannot pack in ordered crystals, or that Rubisco holoenzymes selectively bind only one type of Rubisco SSu.

### 4.5. Rubisco sequence and structural similarity   

Rubisco subunit sequence similarity, especially for the SSus, was not a strong indicator of structural similarity (Table 2 ▸), but the refinement method used for, and the resolution of, the available Rubisco crystal structures may limit the utility of small differences in least-squares deviations to reflect structural similarity. It is also worth noting that the largest structural differences occur when comparing the activated ligand-bound *A. thaliana* Rubisco structure with non-activated Rubisco enzymes (*Halothiobacillus neapolitanus* and *Galdiera sulphuraria* in Table 2 ▸). Overall protein structure can be conserved even when sequence is not; however, small sequence changes can give rise to ‘disproportionate’ or unexpected changes to enzyme structure–function (Wood & Pearson, 1999 ▸). An absence of a direct correlation between primary amino-acid sequence and structure may contribute to the lack of success using *in silico* methods to predict the effect of sequence changes on Rubisco structure and function (Whitney *et al.*, 2011 ▸).

### 4.6. Alternative *A. thaliana* SSu isoforms: functional importance?   

Differential expression of alternative SSu isoforms provides the opportunity for Rubisco regulation. Whether this regulation is strictly to control total Rubisco content, or also allows kinetic alterations, has yet to be resolved. Whilst studies suggest that the expression of multiple SSu isoforms provides a mechanism for the tight control of the total Rubisco content within the chloroplast (Izumi *et al.*, 2012 ▸; Atkinson *et al.*, 2017 ▸), certain SSu isoforms may result in higher substrate specificity under elevated temperatures (Cavanagh, 2016 ▸).

A Rubisco with a higher specificity for CO_2_ than for O_2_ (*i.e.* with a higher *S*_c/o_) would reduce photorespiration and its associated loss of fixed CO_2_ and energy costs. This is envisaged to be of particular benefit in (i) shaded leaves where photosynthesis is primarily limited by electron transport (Long *et al.*, 2006 ▸) and (ii) at higher temperatures where the oxygenation reaction is favoured because of its higher activation energy and reduced CO_2_ solubility (relative to O_2_ solubility; Ku & Edwards, 1977 ▸; Chen & Spreitzer, 1992 ▸). Transcript levels of the *rbc*SB family increase with higher temperatures (Yoon *et al.*, 2001 ▸). Thus, the higher substrate specificity that may be conferred by the RbcS3B isoform (Cavanagh, 2016 ▸) would be advantageous to the plant under an environmental condition that induces transcription of this SSu isoform. There are only two subtle amino-acid differences between the RbcS1B and RbcS2B/RbcS3B mature peptides (Fig. 2 ▸). If RbcS1B confers similar kinetic properties to RbcS3B, then its expression pattern would also be advantageous to the plant: *rbc*S1B is almost exclusively expressed on the abaxial side of leaves (*i.e.* under light-limiting conditions; Sawchuk *et al.*, 2008 ▸).

The structural data presented in this study do not reveal whether alternative *A. thaliana* Rubisco SSus could influence holoenzyme kinetics. However, it is not inconceivable that amino-acid variations in different SSus, such as that observed between the RbcSB family and RbcS1A, could influence *A. thaliana* Rubisco kinetics (Fig. 2 ▸ and Supplementary Table S1). Various mutations in the SSu of *Chlamydomonas reinhardtii* Rubisco can alter holoenzyme kinetic performance (Genkov & Spreitzer, 2009 ▸), and even seemingly subtle mutations in Rubisco can have dramatic functional effects (Whitney *et al.*, 2011 ▸). Amino-acid differences between the *A. thaliana* Rubisco SSu isoforms can be found in regions that have been shown to influence Rubisco kinetics in other organisms, such as the N-terminus and the βA–βB loop. Mutations in the SSu N-terminus influence kinetics (Kostov *et al.*, 1997 ▸), and residues within the Rubisco SSu βA–βB loop and the structurally equivalent loop in non-green algal Rubiscos (named βE–βF) that line the solvent channel and contact Rubisco LSus are known to influence Rubisco kinetics, particularly *S*_c/o_ (Karkehabadi *et al.*, 2005 ▸; Spreitzer *et al.*, 2005 ▸). Furthermore, many of the variable sites are close to the LSu helix α8. Interactions between the SSu and the LSu helix α8 have been proposed to influence Rubisco catalysis (Genkov & Spreitzer, 2009 ▸).

## 5. Related literature   

The following references are cited in the Supporting Information for this article: Felsenstein (1985 ▸), Nei & Kumar (2000 ▸), Rzhetsky & Nei (1992 ▸), Saitou & Nei (1987 ▸) and Tamura *et al.* (2013 ▸).

## Supplementary Material

PDB reference: Rubisco from *Arabidopsis thaliana*, 5iu0

Supplementary Figures and Table.. DOI: 10.1107/S2059798317017132/nj5272sup1.pdf

## Figures and Tables

**Table 1:** Data-collection and refinement statistics Values in parentheses are for the highest resolution shell.

Data collection	
X-ray source	ID29, ESRF, Grenoble
Wavelength (Å)	0.978
Space group	*I*4
Unit-cell parameters (Å)	*a* = *b* = 111.9, *c* = 197.7
Resolution (Å)	1.5
No. of unique reflections	192721
Completeness (%)	99.6 (98.4)
*R*_meas_ †	0.101 (0.742)
〈*I*/σ(*I*)〉	8.9 (1.8)
CC_1/2_	99.4 (43.9)
Refinement statistics	
Residues in model	*A*13–*A*475, *I*1–*I*123, *B*12–*B*475, *J*1–*J*123
No. of solvent molecules	1017
No. of ethylene glycol molecules	14
No. of 2-CABP molecules	2
No. of Mg^2+^ ions	2
Twin fraction	0.48
*R*_cryst_ ‡	0.136
*R*_free_ §	0.152
R.m.s.d., bond lengths (Å)	0.006
R.m.s.d., angles (°)	0.877

† As defined by Diederichs & Karplus (1997 ▸).

‡ *R*_cryst_ = 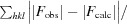 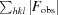, where *F*_obs_ and *F*_calc_ are the observed and calculated structure-factor amplitudes, respectively.

§ *R*_free_ was calculated from a randomly selected 5% of unique reflections.

**Table 2:** Comparison of available L_8_S_8_ Rubisco structures A pairwise evaluation of the sequence and structural homology between *A. thaliana* Rubisco and all L_8_S_8_ Rubiscos with known crystal structures. Structural superpositions were performed with PDB entries 5iu0 (*A. thaliana*; this work), 4hhh (*Pisum sativum*; Loewen *et al.*, 2013 ▸), 4rub (*Nicotiana tabacum*; Suh *et al.*, 1987 ▸), 1wdd (*Oryza sativa*; Matsumura *et al.*, 2012 ▸), 8ruc (*Spinacia oleracea*; Andersson, 1996 ▸), 1gk8 (*Chlamydomonas reinhardtii*; Taylor *et al.*, 2001 ▸), 3zxw (*Thermosynechococcus elongatus*; B. Terlecka, V, Wilhelmi, W. Bialek, B. Gubernator, A. Szczepania & E. Hofmann, unpublished work), 1rbl (*Synechococcus* sp. 6301; Newman *et al.*, 1993 ▸), 1svd (*Halothiobacillus neapolitanus*; C. A. Kerfeld, M. R. Sawaya, I. Pashkov, G. Cannon, E. Williams, K. Tran & T. O. Yeates, unpublished work), 4f0k (*Galdieria sulphuraria*; Stec, 2012 ▸), 1bwv (*Galdieria partita*; Sugawara *et al.*, 1999 ▸) and 1bxn (*Alcaligenes eutrophus*; Hansen *et al.*, 1999 ▸), using the chains indicated in the table. The LSu and SSu *A. thaliana* sequences used for sequence comparisons were NP_051067.1 and BAB09355.1 (Rbcs1B), respectively. Unless otherwise indicated, all structures included in the comparison are of the activated Rubisco complex with 2-CABP bound.

				Chain ID		Sequence similarity to *A. thaliana* Rubisco		LSu		SSu	
Organism	Lineage	Rubisco form	PDB code	LSu	SSu	LSu (%)	SSu (%)	No. of aligned residues	R.m.s.d. (Å)	No. of aligned residues	R.m.s.d. (Å)
Pea†	Higher plant	1B	4hhh	*A*	*S*	95	75	456	0.43	122	0.54
Tobacco	Higher plant	1B	4rub	*A*	*S*	94	74	460	0.38	121	0.48
Rice	Higher plant	1B	1wdd	*A*	*S*	94	72	463	0.25	122	1.20
Spinach	Higher plant	1B	8ruc	*A*	*I*	93	76	463	0.30	121	0.53
Chlamydomonas	Green alga	1B	1gk8	*A*	*I*	88	49	463	0.33	119	0.88
*T. elongatus*	Cyanobacterium	1B	3zxw	*A*	*B*	82	46	462	0.39	93	0.79
*Synechococcus* sp. 6301	Cyanobacterium	1B	1rbl	*A*	*I*	82	43	463	0.34	108	0.85
*H. neapolitanus* ‡	Proteobacterium	1A	1svd	*A*	*M*	75	30	435	0.77	106	1.13
*G. partita*	Non-green alga	1D	1bwv	*A*	*S*	59	34	463	0.72	100	1.32
*G. sulphuraria* ‡	Non-green alga	1D	4f0k	*A*	*B*	59	34	432	1.10	100	1.39
*A. eutrophus* ‡	Proteobacterium	1C	1bxn	*A*	*I*	59	31	441	0.95	97	1.22

† Activated complex with RuBP.

‡ Non-activated complex.
