# Supplementary material for: Structure of Rubisco from Arabidopsis thaliana in complex with 2-carboxyarabinitol-1,5-bisphosphate
Source: Acta Crystallogr D Struct Biol. 2018 Jan 1;74(Pt 1):1–9. doi: 10.1107/S2059798317017132 (PMC5786004; doi:10.1107/S2059798317017132)
Supplement: Supplementary file 1 [file d-74-00001-sup1.pdf]

Acta Cryst D STRUCTURAL BIOLOGY

Volume 73 (2017)

Supporting information for article:

Structure of Rubisco from *Arabidopsis thaliana* in complex with 2-carboxyarabinitol-1,5-bisphosphate

Karin Valegård, Dirk Hasse, Inger Andersson and Laura H. Gunn

Table S1 The location and nature of amino acid differences between the *A. thaliana* Rubisco SSu isoforms

Residue positions that differ between the isoforms are only included for the mature SSu peptide. See Fig. 2 and Fig. S1 for descriptions of SSu and LSu secondary structure features, respectively.

	Amino acid		Location			
Residue #	RbcS3	RbcS1A	In SSu	In holoenzyme structure	Proximal to	Nature of change
2	Lys	Gln	N-terminus	Solvent exposed, packs against LSu	LSu helix α8	Charged/uncharged residue
					LSu helix αK	Potential ionic influence on LSu Glu454 in RbcS3 SSus
22	Thr/Ser	Thr	N-terminal to helix αA	Solvent exposed	SSu helix αA is near LSu helix α8	Minor difference in side chain
24	Val	Ser	Helix αA	Solvent exposed		Hydrophobic/polar
34	Leu	Ile	Helix αA	Solvent exposed		Minor difference in side chain
58	Thr	Ser	Apex of βA-βB loop	Solvent channel	LSu helix α3 in two different LSus	Minor difference in side chain
96	Gly	Asn	Loop αB-βC	Solvent exposed	SSu C-tail	Glycine / polar side chain
					Start of SSu βA-βB loop	
124	Asp/Glu	Gly	C-terminus	Not resolved in structure		Charged /uncharged residue
125	Ala	-	C-terminus	Not resolved in structure		Absent in RbcS1A

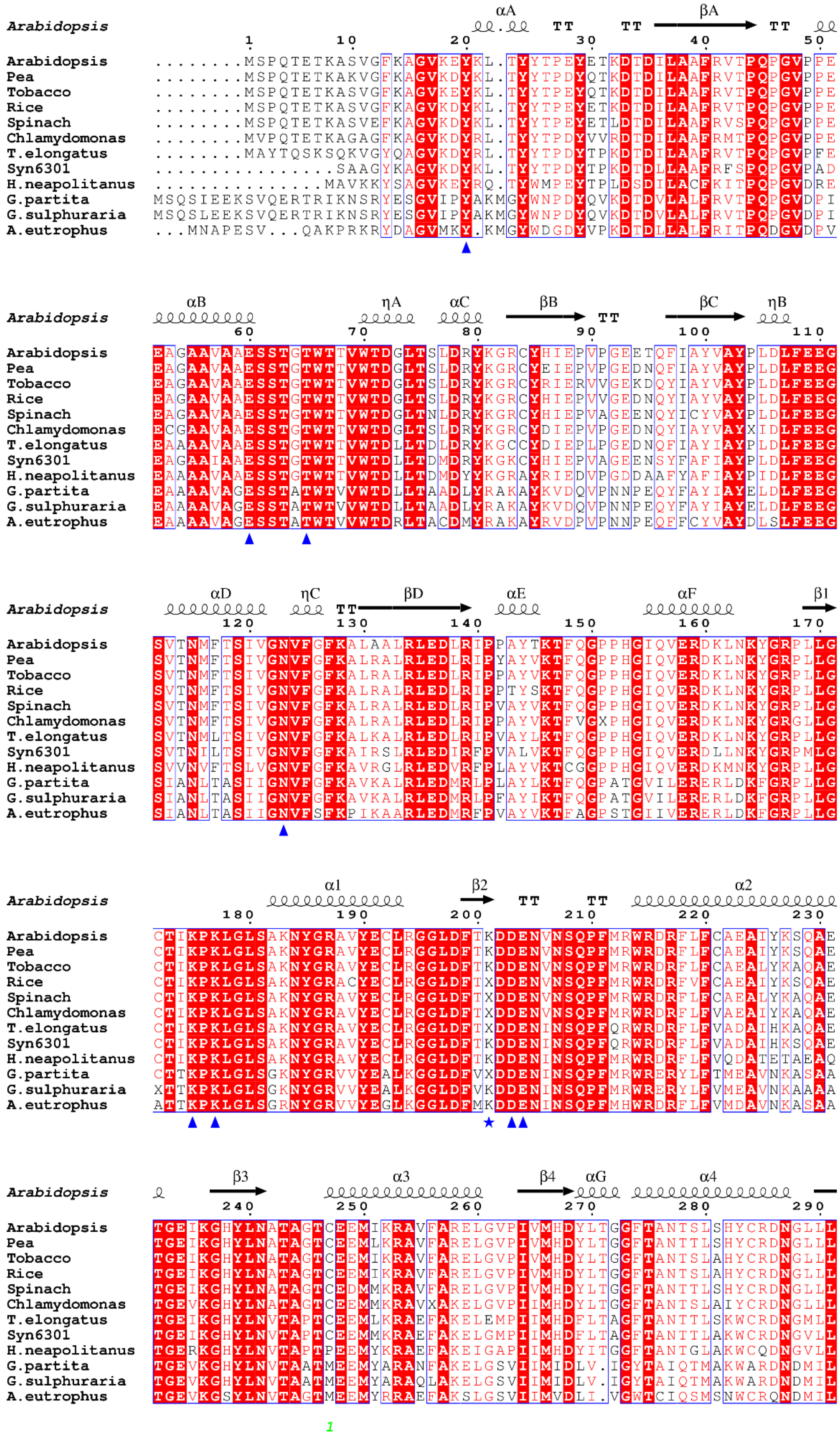
Arabidopsis
Pea
Tobacco
Rice
Spinach
Chlamydomonas
T.elongatus
Syn6301
H.neapolitanus
G.partita
G.sulphuraria
A.eutrophus

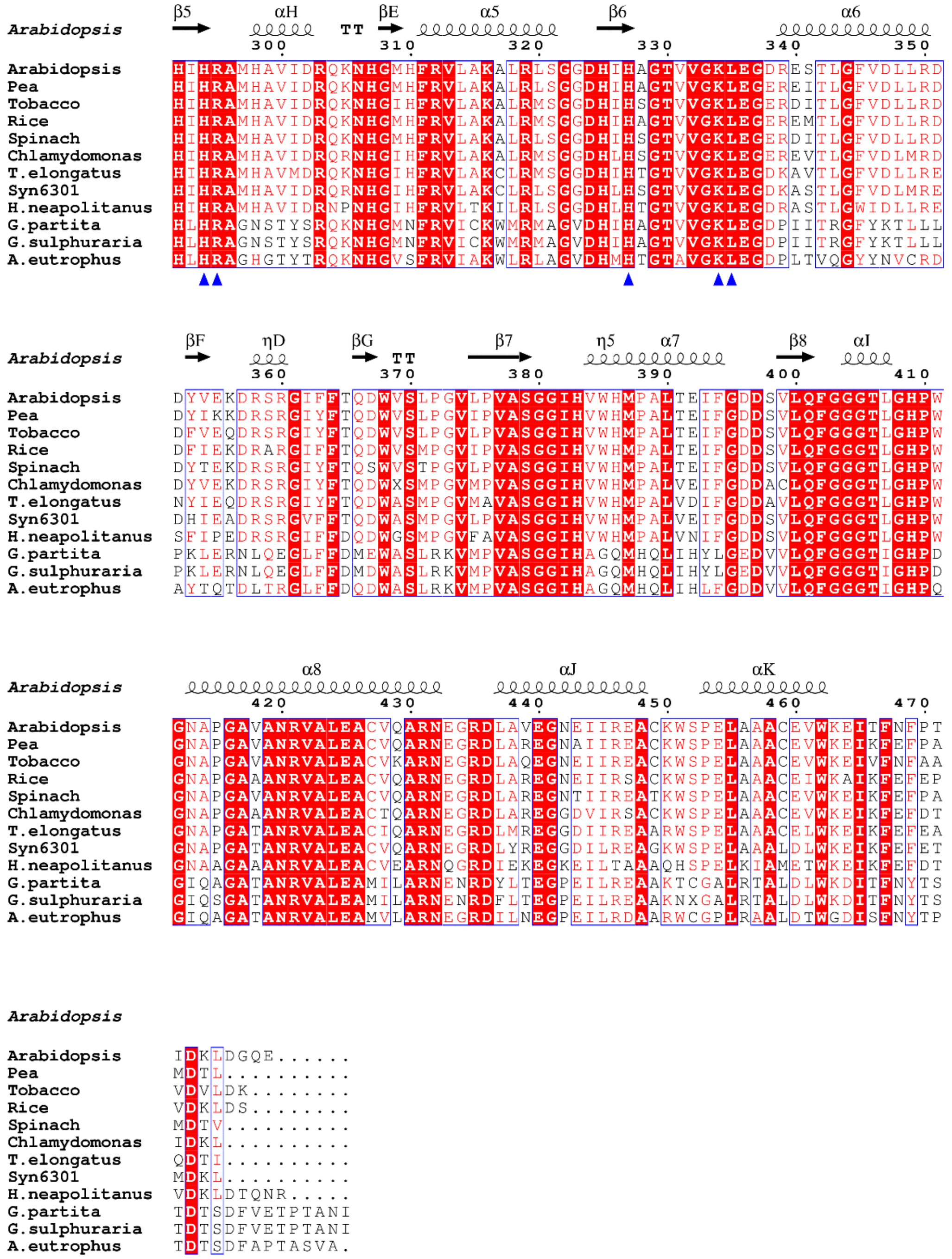

Figure S1 Structure-based sequence alignment of Rubisco LSu sequences. See Fig. 2 figure legend for a description of residue colours. Residue numbering along the top refers to *A. thaliana* Rubisco. *Symbols above* blocks of sequences annotate the Rubisco LSu secondary structure from coordinates 5IU0: α, α-helix; η, 310- helix; β, β-strand; TT, tight β-turns. *Symbols below* blocks of sequences indicate the location of residues that contribute to the active site (blues triangles), the catalytic lysine residue (blue star). The Rubisco LSu structural features are labelled according to convention (Knight *et al.*, 1990), where secondary

structure elements are named βA, βB..., αA, αB... etc, except for the 8 C-terminal βα barrel units, which are numbered β1, β2..., α1, α2... etc. The sequence alignment was created using the accession numbers 4HHH_A (Pea), 4RUB_A (Tobacco), 1WDD_A (Rice), 8RUC_A (Spinach), 1GK8_A (Chlamydomonas), 3ZXW_A (*T. elongatus*), 1RBL_A (*Synechococcus* sp. 6301), 1SVD_A (*H. neapolitanus*), 1BWV_A (*G. partita*), 4F0K_A (*G. sulphuraria*) and 1BXN_A (*A. eutrophus*).

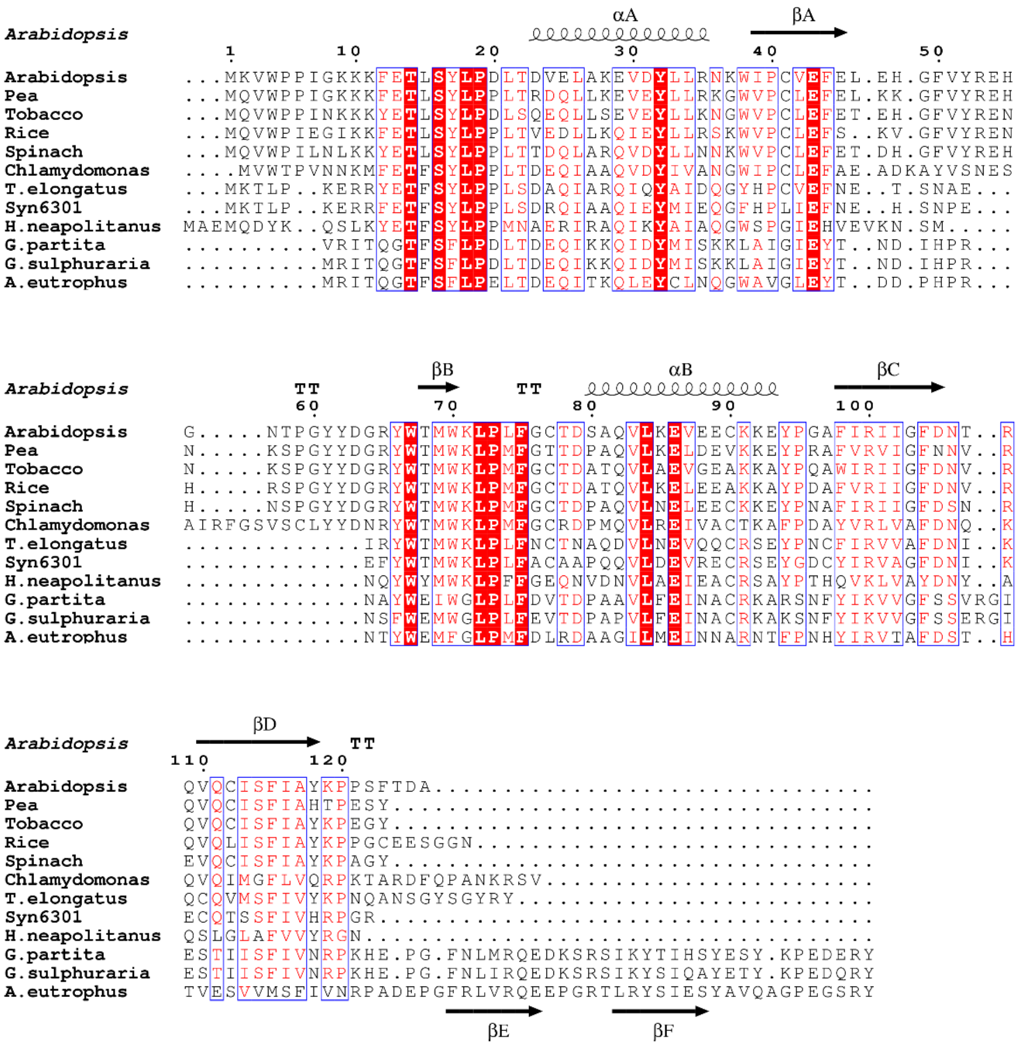

Figure S2 **Structure-based sequence alignment of Rubisco SSu sequences.** See Fig. 2 figure legend for a description of symbols and residue colours. *Symbols above* blocks of sequences annotate the Rubisco SSu secondary structure elements (αA, αB, βA, βB, βC and βD; Knight *et al.*, 1990) from coordinates 5IU0. *Symbols below* blocks of sequences indicate the location of the βE and βF sheets (from co-ordinates 1BWV) unique to form IC and ID Rubiscos. The sequence alignment was created using the accession numbers 4HHH_S (Pea), 4RUB_S (Tobacco), 1WDD_S (Rice), 8RUC_I (Spinach), 1GK8_I (Chlamydomonas), 3ZXW_B (*T. elongatus*), 1RBL_I (*Synechococcus* sp. 6301), 1SVD_M (*H. neapolitanus*), 1BWV_S (*G. partita*), 4F0K_B (*G. sulphuraria*) and 1BXN_I (*A. eutrophus*).

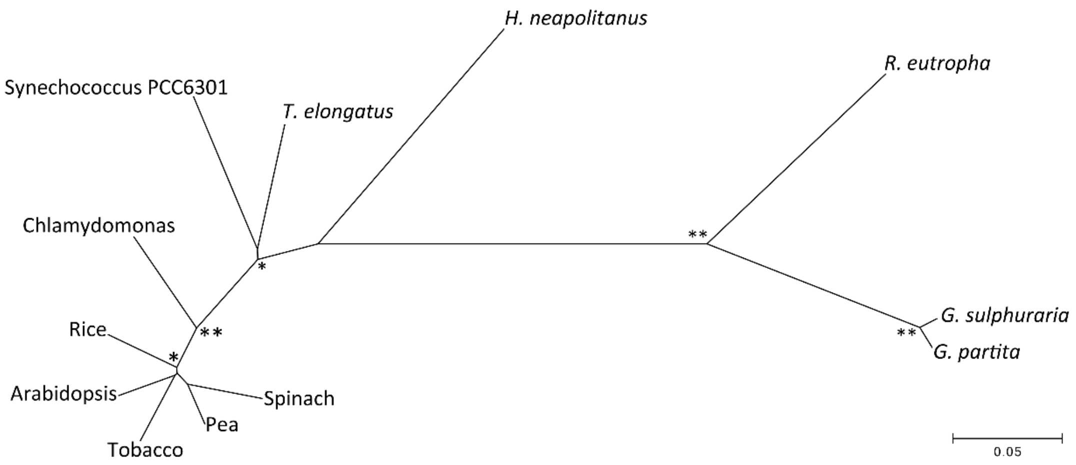

Figure S3 Evolutionary relationships of Rubisco LSus. The evolutionary history was inferred using the Minimum Evolution method (Rzhetsky & Nei, 1992). The optimal tree with the sum of branch length = 0.98484718 is shown. Replicate trees in which the associated taxa clustered together in ≥95% (** = 100%, * = 99%) of the bootstrap iterations (1000 replicates) are shown at branch points (Felsenstein, 1985). The tree is drawn to scale, with branch lengths in the same units as those of the evolutionary distances used to infer the phylogenetic tree. The evolutionary distances were computed using the p-distance method (Nei & Kumar, 2000) and are in the units of the number of amino acid differences per site. The ME tree was searched using the Close-Neighbor-Interchange (CNI) algorithm (Nei & Kumar, 2000) at a search level of 1. The Neighbor-joining algorithm (Saitou & Nei, 1987) was used to generate the initial tree. The analysis involved 12 amino acid sequences. All positions containing gaps and missing data were eliminated. There were a total of 455 positions in the final dataset. Evolutionary analyses were conducted in MEGA6 (Tamura *et al.*, 2013).

```
1B      MASSMLSSAA VVTSPAQATM VAPFTGLKSS ASFPVTRKAN NDITSITSNG [ 50]
2B      .....F..T. .......... .......... .......... .......... [ 50]
3B      .......... .......... .......... .A......T. K.....A... [ 50]
1A      .........T M.A....... ....N..... .A..A..... .......... [ 50]
5IU0    ---------- ---------- ---------- ---------- ---------- [ 50]

1B      GRVSCMKVWP PIGKKKFETL SYLPDLTDVE LAKEVDYLLR NKWIPCVEFE [100]
2B      .......... .......... ......S... .......... .......... [100]
3B      .......... .......... ......S... .......... .......... [100]
1A      ...N..Q... .......... ........S. ........I. .......... [100]
5IU0    -----..... .......... .......... .......... .......... [100]

1B      LEHGFVYREH GNTPGYYDGR YWTMWKLPLF GCTDSAQVLK EVEECKKEYP [150]
2B      .......... .......... .......... .......... .......... [150]
3B      .......... .......... .......... .......... .......... [150]
1A      .......... ..S....... .......... .......... .......... [150]
5IU0    .......... .......... .......... .......... .......... [150]

1B      GAFIRIIGFD NTRQVQCISF IAYKPPSFTD A [181]
2B      .......... .......... .........E . [181]
3B      .......... .......... .........E . [181]
1A      N......... .......... .........G - [181]
5IU0    .......... .......... ........-- - [181]
```

Figure S4 Sequence alignment of the *A. thaliana* Rubisco SSu precursors. The *A. thaliana* Rubisco structure presented in this study (pdb 5IU0) contains the RbcS1B isoform. Residues are numbered relative to the precursor peptide sequence. Identity is represented by dots, and gaps by dashes, and the transit peptide is shaded grey. See Fig. 2 figure legend for accession numbers.

Supplementary references

Diederichs, K. & Karplus, P. A. (1997). *Nat. Struct. Biol.* **4**, 269–275.

Felsenstein, J. (1985). *Evol. Int. J. Org. Evol.* **39**, 783–791.

Nei, M. & Kumar, S. (2000). Molecular Evolution and Phylogenetics Oxford University Press.

Rzhetsky, A. & Nei, M. (1992). *Mol. Biol. Evol.* **9**, 945.

Saitou, N. & Nei, M. (1987). *Mol. Biol. Evol.* **4**, 406–425.

Tamura, K., Stecher, G., Peterson, D., Filipski, A. & Kumar, S. (2013). *Mol. Biol. Evol.* **30**, 2725–2729.
